# CD146^+^ mural cells from infantile hemangioma display proangiogenic ability and adipogenesis potential *in vitro* and in xenograft models

**DOI:** 10.3389/fonc.2023.1063673

**Published:** 2023-04-27

**Authors:** Jialin Chen, Qianyi Chen, Yajing Qiu, Lei Chang, Zhang Yu, Yuanbo Li, Shih-jen Chang, Zongan Chen, Xiaoxi Lin

**Affiliations:** The Department of Plastic and Reconstructive Surgery, Shanghai Ninth People’s Hospital, Shanghai Jiao Tong University School of Medicine, Shanghai, China

**Keywords:** infantile hemangioma, mural cell, CD146, xenograft model, angiogeneis, adipogenensis

## Abstract

**Objective:**

Infantile hemangioma (IH), the most common infantile vascular neoplasm, is uniquely characterized by rapid proliferation followed by slow spontaneous involution lasting for years. In IH lesions, perivascular cells are the most dynamic cell subset during the transition from the proliferation phase to the involution phase, and we aimed to systematically study this kind of cell.

**Methods and results:**

CD146-selective microbeads were used to isolate IH-derived mural-like cells (HemMCs). Mesenchymal markers of HemMCs were detected by flow cytometry, and the multilineage differentiation potential of HemMCs was detected by specific staining after conditioned culture. CD146-selected nonendothelial cells from IH samples showed characteristics of mesenchymal stem cells with distinct angiogenesis-promoting effects detected by transcriptome sequencing. HemMCs spontaneously differentiated into adipocytes 2 weeks after implantation into immunodeficient mice, and almost all HemMCs had differentiated into adipocytes within 4 weeks. HemMCs could not be induced to differentiate into endothelial cells *in vitro*. However, 2 weeks after implantation *in vivo*, HemMCs in combination with human umbilical vein endothelial cells (HUVECs) formed GLUT1^+^ IH-like blood vessels, which spontaneously involuted into adipose tissue 4 weeks after implantation.

**Conclusions:**

In conclusion, we identified a specific cell subset that not only showed behavior consistent with the evolution of IH but also recapitulated the unique course of IH. Thus, we speculate that proangiogenic HemMCs may be a potential target for the construction of hemangioma animal models and the study of IH pathogenesis.

## 1 Introduction

Infantile hemangioma (IH) is the most common infantile vascular neoplasm, with a prevalence of 3%–10% in neonates (1). The disease is characterized by rapid proliferation in the first few weeks of life followed by slow spontaneous involution lasting for years. Growth is usually rapid during the first 6 months of the proliferative phase, especially within 3 months. The growth phase may extend until the 6th to 9th month at low speed. The involuting phase begins at approximately 1 year of age and continues for 3–7 years (1, 2). Six months to 1 year old were considered stable stage or late proliferation. Although oral propranolol has been proven clinically effective in treating infantile hemangioma, the mechanism of rapid angiogenesis initiation and spontaneous involution remains largely unknown (3, 4).

The progression of infantile hemangioma involves various cells, including hemangioma-derived mesenchymal stem cells (HemMSCs), stem cells (expressing CD133, HemSCs), endothelial cells (expressing CD31, HemECs), pericytes and vascular smooth muscle cells (VSMCs) (5–8). CD133-selected HemSCs are considered the cellular origin of infantile hemangioma, exhibiting a special phenotype similar to that of mesenchymal stem cells (MSCs) and the potential for *de novo* formation of blood vessels (7, 9). Recent studies have indicated that hemangioma pericytes, similar to HemSCs, have MSC-like features and may be an important source of fibrofatty tissue during involution (10). Mural cells, comprising pericytes and VSMCs, are perivascular cells embracing endothelial cells (11, 12)and have been recognized as a specific cell cluster of MSCs (13). In preliminary results from our research group, through single-cell RNA sequencing (scRNA-seq), we found that the most variable cell population during the transition from the proliferation phase to the involution phase was hemangioma mural cells (HemMCs). We also identified that cluster of differentiation 146 (CD146) can serve as a cell marker to distinguish HemMCs. Perivascular cells of proliferating IH tissue have been stained positive for neural glial antigen-2 (NG2), platelet-derived growth factor receptor-(PDGFR)-β, calponin I, α-smooth muscle actin (αSMA) and smooth muscle myosin heavy chain (smMHC) (5). Neither marker is specific to perivascular cells: PDGFRβ is expressed by fibroblasts, αSMA is expressed by myofibroblasts, and NG2 is expressed by oligodendrocyte progenitor cells (14).

CD146, also known as melanoma cell adhesion molecule (MCAM), is a transmembrane glycoprotein that acts not only as an adhesion molecule but also as a cellular surface receptor of miscellaneous ligands (15). CD146 is highly expressed in embryonic tissues but weakly expressed in normal adult tissues, and its expression is frequently increased in rapidly proliferating cells (16). Despite its role as an adhesion molecule, CD146 can act as a coreceptor for vascular endothelial growth factor receptor 2 (VEGFR2) to participate in angiogenesis and a receptor for growth factors to participate in cell growth, proliferation, differentiation, and survival (15). In adult human tissues, CD146 is expressed in mural cells of blood microvessels and is considered a pericyte marker (17). In a previous study, strong expression of CD146 was observed in pericyte-like cells residing in hemangiomas (18). Anti-CD146 antibody-conjugated microbeads were previously used to isolate this kind of cell (19), but their function remained unknown. Thus, we aimed to investigate the phenotype and function of hemangioma-derived CD146^+^ mural cells and their role in hemangiomas.

In this study, we isolated CD31^-^CD146^+^ HemMCs from proliferative IH specimens using antibody-conjugated microbead kits and investigated the phenotype and function of the cells *in vitro* and *in vivo* to explore their role in the proliferation and involution of IH and to establish a new experimental model for studying IH.

## 2 Materials and methods

### 2.1 Cell isolation and culture

All cells were isolated from three proliferative hemangiomas from three patients less than 6 months old. IH samples were placed in 50-mL sterile tubes containing phosphate-buffered saline immediately after excision and stored at 4°C. Cell suspensions were obtained by incubating minced specimens in endothelial cell growth medium 2 (EGM-2, PromoCell) supplemented with 2 mg/ml collagenase (Sigma Aldrich), 0.02 mg/ml DNase (Sigma Aldrich), 10% fetal bovine serum (Thermo Fisher Scientific) and 1% penicillin–streptomycin. HemSCs and HemECs were separated by magnetic activated cell sorting (MACS) using a CD133 MicroBead Kit (Miltenyi Biotec) and CD31 MicroBead Kit (Miltenyi Biotec), respectively, from a single-cell suspension as previously described (7, 20). Next, HemMCs were separated by CD146-positive selection from CD133^-^CD31^-^ cells using a CD146 MicroBead Kit (Miltenyi Biotec). After separation, HemMCs were cultured in StemPro™ MSC serum-free medium (Thermo Fisher Scientific), and HemSCs were cultured in EGM-2 supplemented with Growth Medium 2 Supplement Mix (PromoCell) and 10% fetal bovine serum. For transcriptional analysis, HemMCs were transferred to complete EGM-2 for 3 days to minimize the influence of the medium. HUVECs were purchased from Promocell and cultured in EGM-2 supplemented with Growth Medium 2 Supplement Mix (PromoCell). All kinds of cells at passages 3–7 were used in the following experiments, including *in vivo* studies.

For mesenchymal differentiation, cells were cultured in adipogenic medium, osteogenic medium and chondrogenic medium (STEMCELL Technologies), and human adipose-derived stem cells (ADSCs), which were isolated and expanded following previously reported protocols (21), were used as a positive control in this study. Endothelial cell differentiation and neurogenic differentiation were induced according to a previous report (7).

To fluorescently label cells, the vector LV051-PHBLV-U6-MCS-EF1-mcherry-T2A-PURO containing the mCherry-encoding sequence was purchased from HanBio Technology. When the culture reached approximately 60% confluence, the HemMCs were infected with Lentivirus carrying the aforementioned vector (multiplicity of infection=10) for 24 hours. Next, the cells were cultured in the complete medium for 3 days. To obtain stable fluorescence-labeled cells, the cells were then screened using puromycin (at the concentration of 2.0 ug/mL for 2 days) and examined by fluorescence microscope (Nikon ECLIPSE microscope).

### 2.2 Flow cytometry analysis

When cell confluence reached 80%-90%, the HemMCs were dissociated using TrypLE™ Express Enzyme (Gibco) and suspended in Cell Staining Buffer (Biolegend). In this study, each test contained 5×10^5^ cells in 100 µL staining buffer. To reduce nonspecific Fc receptor (FcR)-mediated binding, 5 µL FcR blocking solution (Biolegend) was applied per test (10 min incubation at room temperature). The fluorescence dye-conjugated primary antibodies (listed in **Supplementary Table 2**) were applied at recommended concentration indicated in the manufacturer’s instruction. After 30 min incubation at 4°C in the dark, the cells were washed using staining buffer (2 mL per test) for 2 times and then suspended in 200 µL staining buffer. The samples were immediately analyzed by the LSRFortessa flow cytometer (BD Biosciences) or CytoFLEX LX flow cytometer (Beckman Coulter) and FlowJo software (BD Biosciences).

### 2.3 RNA isolation and transcriptome sequencing

Total RNA was isolated from HemMCs and HemSCs using TRIzol and the RNA Nano 6000 Assay Kit with the Bioanalyzer 2100 system. After cDNA library preparation, clustering of the index-coded samples was performed on a cBot Cluster Generation System using TruSeq PE Cluster Kit v3-cBot-HS (Illumina). The library preparations were sequenced on the Illumina NovaSeq platform, and 150 bp paired-end reads were generated (Novogene Experimental Department). Hisat2 v2.0.5 was used for mapping. Feature Counts v1.5.0-p3 was used to count the read numbers mapped to each gene. Then, the FPKM of each gene was calculated based on the length of the gene and read count mapped to this gene. Differential expression analysis between two conditions/groups (three biological replicates per condition) was performed using the DESeq2 R package (1.20.0). The resulting P values were adjusted using Benjamini and Hochberg’s approach for controlling the false discovery rate. For differential expression analysis of two groups, genes with an adjusted P value <0.05 according to DESeq2 were considered differentially expressed. The data generated in this study has been deposited in NCBI’s Gene Expression Omnibus and is accessible through GEO Series accession number GSE216867.

### 2.4 Xenograft model of IH

The murine model of IH was established according to an adapted protocol of Boscolo et al (10). We used female BALB/c nude mice in this study. A total of 1×10^6^ cells (per implant), which were HemMCs or HUVECs alone or the mixture of them at a 1:1 ratio, were suspended in 100 µL medium, mixed thoroughly with 100 µL Matrigel (Corning). Then, the cell/Matrigel mixture (200 µL per implant) was injected subcutaneously onto the back of mice aged 3 weeks (n = 6/group). At 1, 2, 4 and 8 weeks after the injection, the animals were sacrificed, and Matrigel plugs were harvested. The implants were fixed overnight in 4% paraformaldehyde at 4°C, and then paraffin-embedded and sectioned.

### 2.5 Immunohistochemical and immunofluorescence analysis

The sections of eighteen paraffin-embedded IH samples were obtained from the Pathology Department of Shanghai Ninth People’s Hospital. These samples were from the patients who were assessed for the necessity of operation (2) and collected *via* surgical treatment in the last ten years at Shanghai Ninth People’s Hospital. Most of the lesions were located in the head or face, and it was difficult to achieve a satisfactory outcome after drug treatment or natural involution. Detailed patient information is listed in **Supplementary Table S1**. The IH sample or Matrigel xenograft explant sections were deparaffinized by xylene and then hydrated in a series of graded alcohol solutions. Antigen retrieval was performed, using the PH 9.0 antigen retrieval buffer (GeneTech), by heating in a water bath at 60°C for 20 min. The sections were permeabilized in 0.1% Triton X-100 (dissolved in phosphate buffered saline (PBS)) for 5 min after they cooled down to room temperature. Next, the sections were blocked in 5% donkey serum (dissolved in PBS) at room temperature for 1 h and then incubated overnight with primary antibodies (details in **Supplementary Table 2**) at 4°C. The antibodies were applied at recommended concentration indicated in the manufacturer’s instructions. Next, the sections were washed with PBS-Tween 20 (PBST) for 3 times. For immunohistochemistry, the detection of the antigens and counterstaining of the nuclei was performed using a REAL EnVision Detection Kit (DAKO) according to the manufacturer’s instructions. Images of the processed sections were taken with a Nikon ECLIPSE microscope. For Immunofluorescence, the sections were incubated with fluorescein-conjugated secondary antibodies (details in **Supplementary Table 2**) for 1 h at room temperature and then mounted in DAPI containing mounting medium (SouthernBiotech). Immunofluorescence (IF) images were taken with a Leica TCS SP2 Acousto-Optical Beam Splitter confocal system.Immunostaining with each antibody was carried out on at least 3 samples from different patients, and representative images are presented.

### 2.6 Statistical analysis

Statistical significance was determined using GraphPad Prism 6 (GraphPad Software, La Jolla, CA, USA). The data were analyzed by ANOVA or Student’s t test, and differences were deemed significant at P<0.05.

### 2.7 Study approval

IH specimens were collected from the Department of Plastic and Reconstructive Surgery of Shanghai Ninth People’s Hospital, Shanghai Jiao Tong University of Medicine. A diagnosis of IH was confirmed by experienced clinicians and pathologists. Written informed consent for use of the specimens was obtained before surgery. All experiments were performed according to protocols reviewed and approved by the Ethics Committee of Shanghai Ninth People’s Hospital, Shanghai Jiao Tong University of Medicine.

## 3 Results

### 3.1 CD146^+^ cells are enriched in proliferative IH specimens

**Figure 1:**
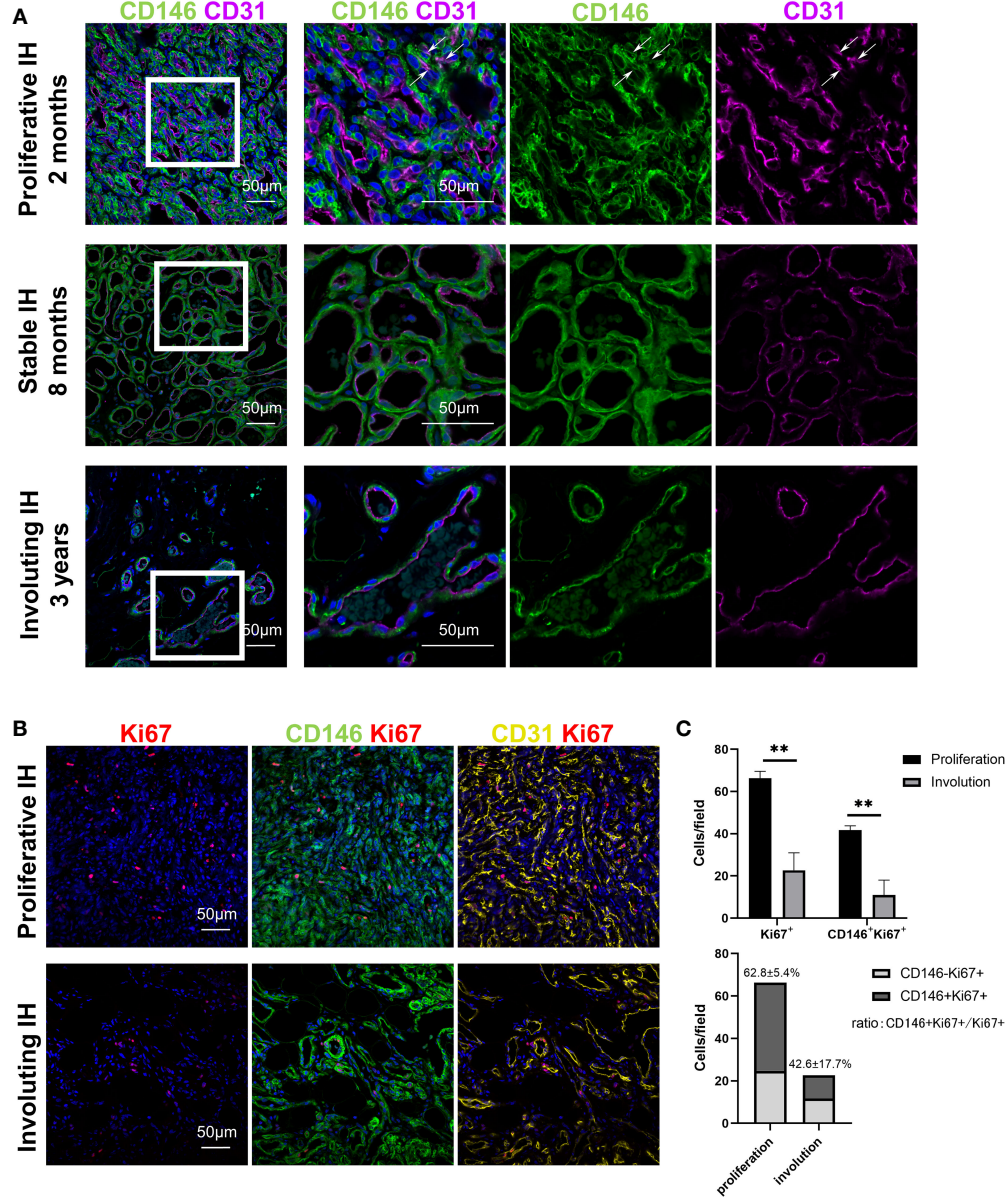
CD146^+^ cells are enriched in proliferative IH specimens. **(A)** Representative images of IH specimens stained for CD146 (green) and CD31 (magenta). A representative proliferative IH specimen was collected from a 2-month-old patient with rapid growth hemangioma. White arrows indicate the CD31^+^ HemECs with CD146 expression. A representative stable IH specimen was collected from an 8-month-old patient with a stable hemangioma. A representative involuting IH specimen was collected from a 3-year-old patient with involuted hemangioma. The results were confirmed in 18 patients. Short scale bars=50 μm; long scale bars=100 μm. **(B)** Representative images of proliferative and involuting IH tissue stained for Ki67 (red), CD146 (green), and CD31 (yellow). **(C)** Quantification of Ki67^+^ and CD146^+^Ki67^+^ cells in proliferating and involuting IH tissue and proportions of CD146^+^Ki67^+^ cells and CD146^-^Ki67^+^ cells in total cells. Scale bars=50 μm. All nuclei were counterstained DAPI (blue). Data are expressed as the mean ± SDM. **, p <0.01. IH, infantile hemangioma.

CD146 expression was measured in specimens from patients with all stages of IH. CD146 expression was mainly located in perivascular cells surrounding CD31^+^ endothelial cells in hemangioma specimens of different phases (**Figure 1A**). In addition, CD146 is expressed in some of the CD31^+^ HemECs in proliferative IH (**Figure 1A**). Many CD146^+^ cells were costained with Ki67^+^ (**Figure 1B**). The number of CD146^+^Ki67^+^ cells was significantly higher in proliferating IH tissues than in involuting IH tissues (**Figure 1C**). The expression of CD146 mostly overlapped with that of PDGFRβ in proliferative hemangioma (**Supplementary Figure 1A**). However, PDGFRβ was also positively expressed in CD31^-^CD146^-^ cells embracing CD146^+^ HemMCs, which were probably telocytes (19) or fibroblasts. PDGFRβ was not expressed in HemMCs in involuting hemangioma (**Supplementary Figure 1A**). Moreover, glucose transporter 1 (GLUT1)-positive HemECs were surrounded by CD146^+^ cells in both the proliferative and involuting phases (**Supplementary Figure 1B**). These results indicated that CD146 was more suitable as a marker to distinguish mural cells of IHs in different phases.

### 3.2 CD146^+^ HemMCs in combination with HemECs survived and proliferated in a xenograft model.

**Figure 2:**
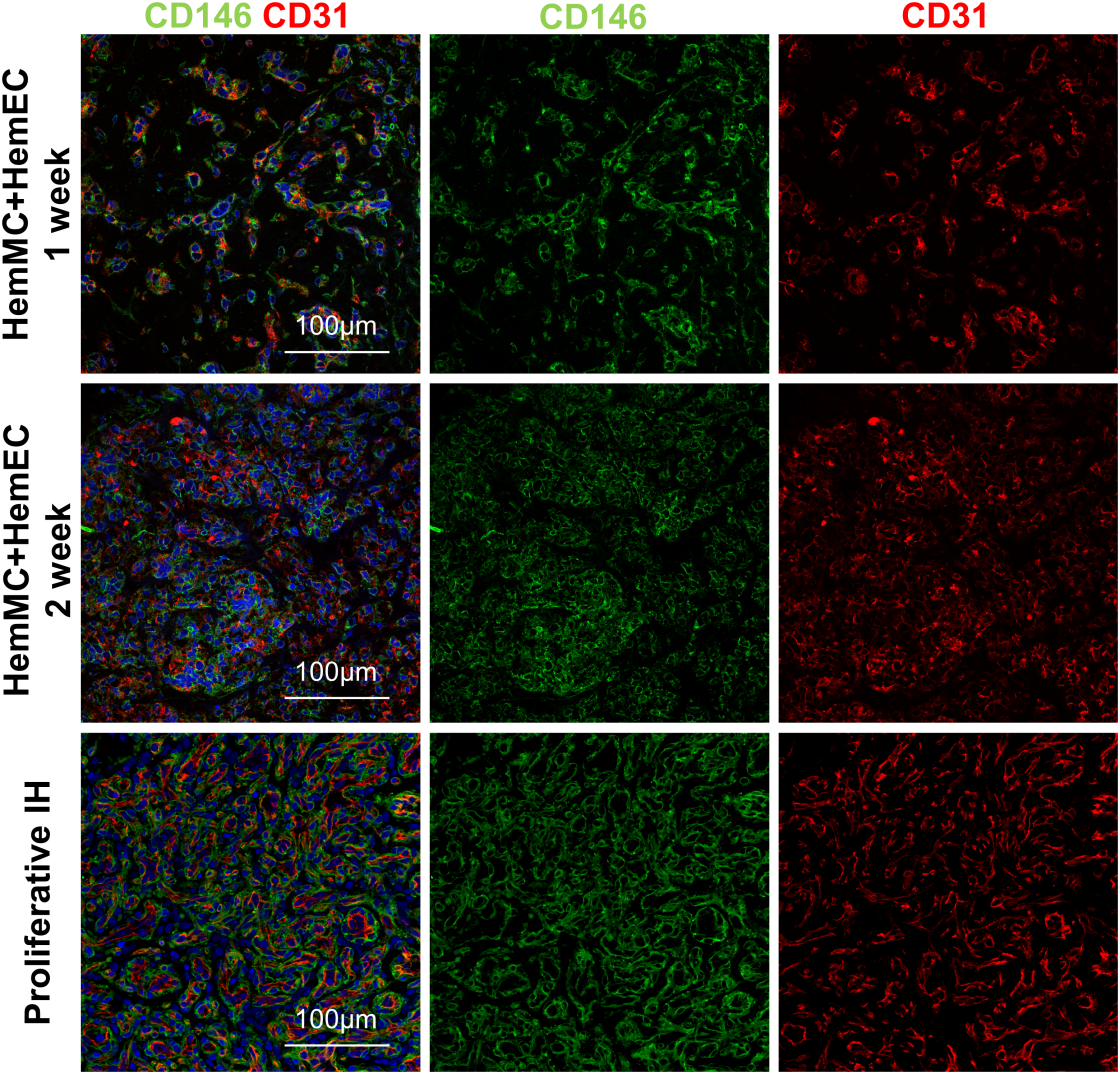
HemMCs and HemECs survive and proliferate in a xenograft model. A mixture of HemECs and HemMCs in Matrigel was injected into nude mice. Explants were harvested at 1 and 2 weeks and then subjected to immunostaining of CD146 and CD31. Scale bars=50 μm. All nuclei were counterstained DAPI (blue). HemMCs, infantile hemangioma mural cells; HemECs, infantile hemangioma endothelial cells.

HemMCs and HemECs were isolated from proliferating hemangioma tissues through MACS and cultured. To assess whether both HemMCs and HemECs survive and proliferate in a xenograft model, a mixture of Matrigel and the two kinds of cells, i.e., HemMCs and HemECs, were injected into immunodeficient nude mice. At 1 week, both CD31^+^ cells and CD146^+^ cells were found in the implants by immunostaining (**Figure 2**). At 2 weeks, the density of both became higher (**Figure 2**). Staining of a proliferative IH section was used as a positive control.

### 3.3 CD146^+^ HemMCs express mesenchymal markers and display multilineage differentiation potential

**Figure 3:**
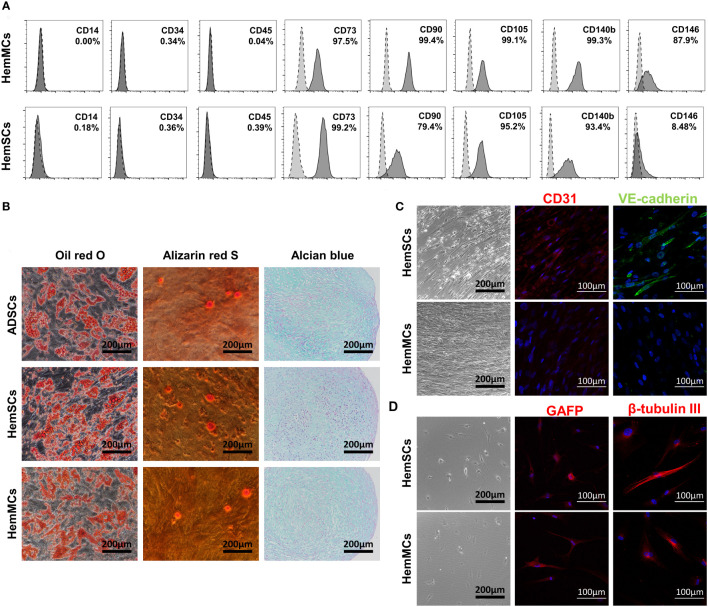
HemMCs express MSC markers and display multilineage differentiation potential. **(A)** Flow cytometric analysis of MSC marker expression in HemMCs and HemSCs. The dark gray histograms show cells labeled with fluorescein-conjugated antibodies. The light gray histograms show the negative control. The percentage of positive cells relative to total cells is shown. **(B)** *In vitro* differentiation of HemMCs into oil red O-stained adipocytes, alizarin red S-stained osteocytes and alcian blue-stained chondrocytes compared with HemSCs. ADSCs were also cultured in the same medium as a positive control. Scale bars=200 μm. **(C)** *In vitro* differentiation of HemMCs into CD31 (red)- or VE-cadherin (green)-positive endothelial cells. Black scale bars=200 μm. White scale bars=100 μm. **(D)** *In vitro* differentiation of HemMCs into GFAP (red)- or β-tubulin III (red)-positive neuroglial cells. Black scale bars=200 μm. White scale bars=100 μm. HemMCs, infantile hemangioma mural cells; MSC, mesenchymal stem cell; HemSCs, infantile hemangioma stem cells; ADSCs, adipose-derived stem cells; VE-cadherin, vascular endothelial cadherin; GFAP, glial fibrillar acidic protein.

To further analyze the phenotypes of HemMCs, flow cytometry was performed to assess the expression of stem cell surface markers. Similar to HemSCs, HemMCs expressed the mesenchymal stem cell surface markers CD73, CD90 and CD105 but did not express CD14, CD34 or CD45 (**Figure 3A**). After culture, the percentage of CD146^+^ cells in HemMCs was maintained at 87.9%, while the percentage of CD146^+^ cells in HemSCs was 8.48%, both counted by flow cytometry (FCM). These results indicated that CD146 was suitable as a marker to distinguish cultured HemMCs. Most HemMCs (97.5%) expressed CD140b (PDGFRβ), which was consistent with our previous results indicating that CD146^+^ cells were localized to the perivasculature (**Figure 3A**). Then, to determine the mesenchymal stem cell differentiation potential of these cells, we cultured HemMCs in conditioned medium and compared them with HemSCs. ADSCs, which are known to have the potential for adipogenesis, osteogenesis and chondrogenesis, were cultured in the same conditioned medium as the positive control. Similar to HemSCs and ADSCs, HemMCs displayed the potential to differentiate into adipocytes, osteocytes, and chondrocytes *in vitro* (**Figure 3B**). HemMCs expressed the neural markers glial fibrillar acidic protein (GFAP) and β-tubulin III after conditioned induction (**Figures 3C, D**). Regarding endothelial differentiation, unlike HemSCs, HemMCs failed to express the EC markers CD31 and vascular endothelial cadherin (VE-cadherin) in the same endothelial differentiation medium.

### 3.4 CD146^+^ HemMCs form adipose tissue *in vivo*

**Figure 4:**
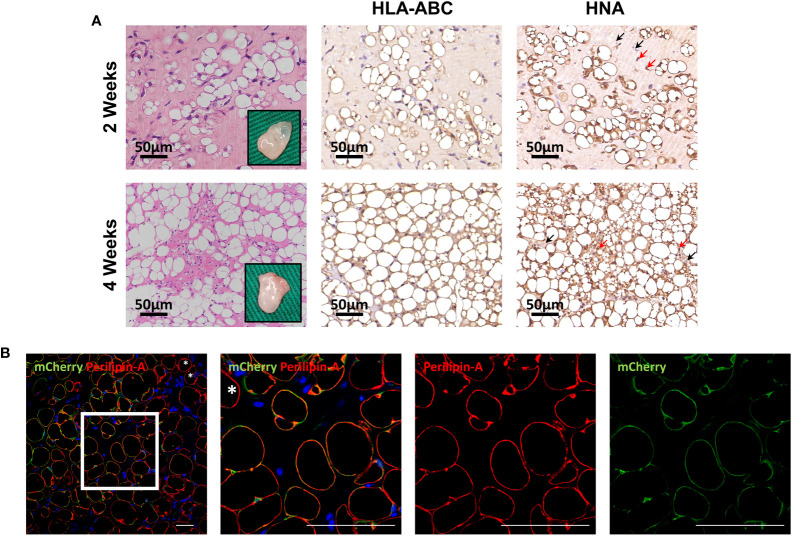
HemMCs form adipocytes *in vivo*. HemMCs or mCherry-labeled HemMCs were mixed with Matrigel and injected into nude mice. **(A)** HemMC/Matrigel implants were harvested at 2 and 4 weeks and then subjected to H&E staining or IHC staining of HLA-ABC and HNA. Scale bars=50 μm. **(B)** mCherry-labeled HemMC/Matrigel implants were harvested at 4 weeks and then subjected to immunofluorescence staining for mCherry (green) and perilipin-A (red). Short scale bars=50 μm; long scale bars=100 μm. Red arrows indicate representative HNA-positive nuclei. Black arrows indicate representative HNA-negative nuclei; * indicates representative mCherry-negative adipocytes. HemMCs, infantile hemangioma mural cells; H&E, hematoxylin and eosin; IHC, immunohistochemistry; HLA-ABC, human leukocyte antigen-ABC. HNA, human nuclei antigen.

To further evaluate differentiation potential *in vivo*, HemMCs were mixed with Matrigel and injected subcutaneously into nude mice. Two weeks or four weeks after injection, the HemMC/Matrigel implants were harvested, sectioned, and stained. Similar to the involuting infantile hemangiomas shown, hematoxylin and eosin (H&E) staining revealed that a portion of the explants formed adipose tissue at 2 weeks. At 4 weeks, many of the explants had turned into fat tissue (**Figure 4A**). IHC staining for human leukocyte antigen-ABC (HLA-ABC) or human nuclei antigen (HNA) indicated that these adipocytes were of a human source (**Figure 4A**). Moreover, mCherry-labeled HemMC/Matrigel explants were stained for perilipin-A, and the results showed that these cells were perilipin-A^+^ adipocytes derived from human cells (**Figure 4B**).

### 3.5 CD146^+^ HemMCs exhibit a proangiogenic transcriptome and function

**Figure 5:**
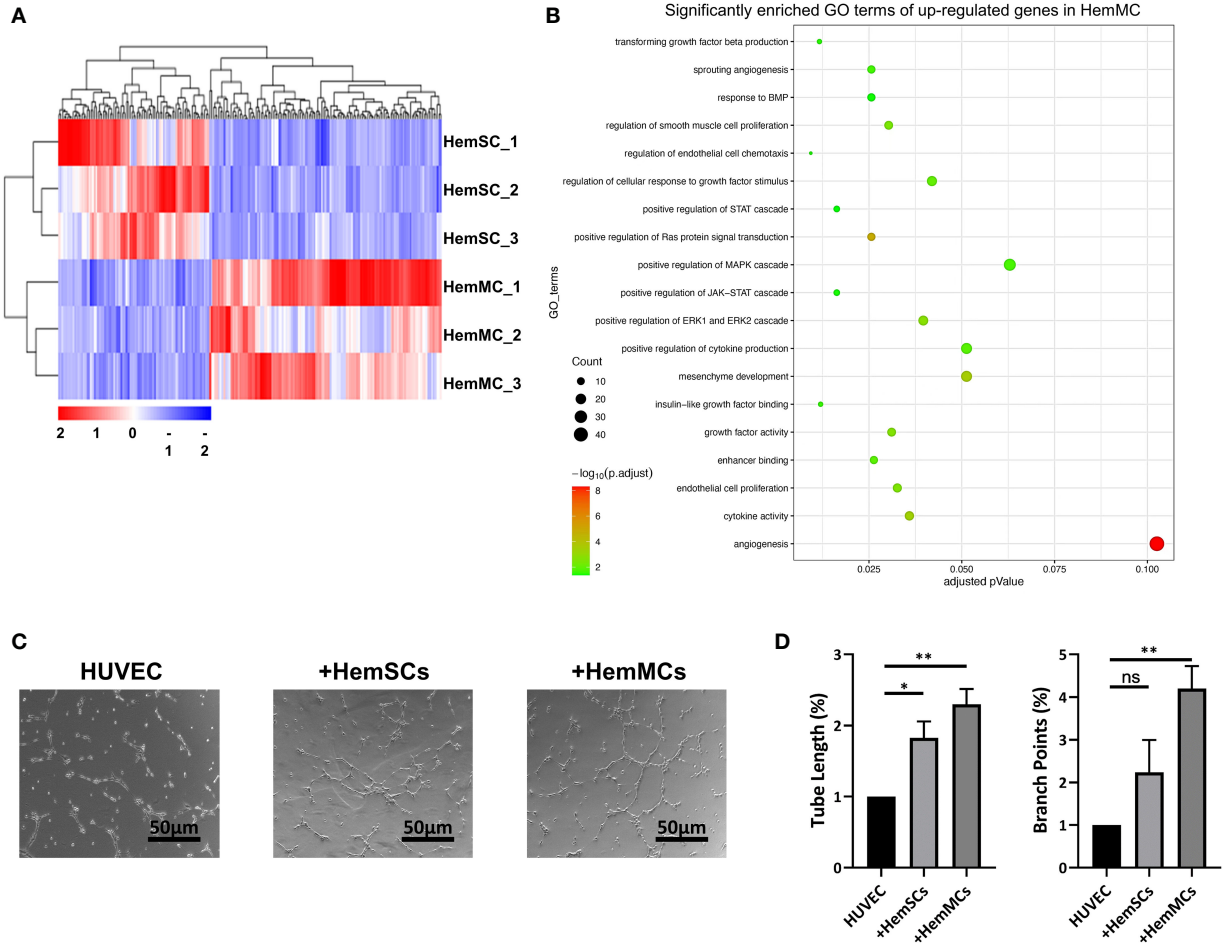
HemMCs display pro-angiogenesis capacity. **(A)** Heatmap showing the transcriptional expression profiles of HemMCs and HemSCs. **(B)** GO enrichment analysis of the differentially expressed genes (the upregulated ones in HemMCs). **(C)** Representative images of tube formation on Matrigel *in vitro* by HUVECs cultured alone, cocultured with HemSCs, or cocultured with HemMCs. Scale bars=50 μm. **(D)** Semiquantitative analysis of the tube length and number of branch points of HUVEC tubes. ImageJ and GraphPad Prism software were used for analysis. *, p <0.05; **, p <0.01; ns, p>0.05. HemMCs, infantile hemangioma mural cells; HemSCs, infantile hemangioma stem cells; GO, gene ontology; HUVECs, human umbilical vein endothelial cells.

To identify the molecular signature of CD146^+^ HemMCs and their different role in IH from HemSCs, we performed RNA sequencing (RNA-seq) and compared the transcriptome between *in vitro* cultured HemMCs and HemSCs. A total of 216 differentially expressed genes, including 131 upregulated genes and 85 downregulated genes, were found between HemMCs and HemSCs (**Figure 5A**). Three biological replicates from each group were subjected to clustered analysis. Gene Ontology (GO) enrichment analysis showed that the upregulated genes in HemMCs were involved in the extracellular matrix and angiogenesis (**Figure 5B**), which implied that HemMCs exhibited a pro-angiogenesis-associated transcriptome.

To confirm the proangiogenic function of HemMCs, HUVECs were seeded on Matrigel to induce tube formation, cultured independently or cocultured with HemMCs or HemSCs. The results showed that the tube length and number of branch points of HUVEC tubes were increased in the HemMC coculture group compared with the HUVEC alone group or HemSC coculture group (**Figures 5C, D**). In addition, significantly enriched GO terms, including growth factor activity (GO: 0008083) and enhancer binding (GO: 0035326), may partly account for the proangiogenic function of HemMCs (**Supplementary Figure 2A, B**).

### 3.6 CD146^+^ HemMCs in combination with HUVECs form GLUT1^+^ blood vessels *in vivo*

**Figure 6:**
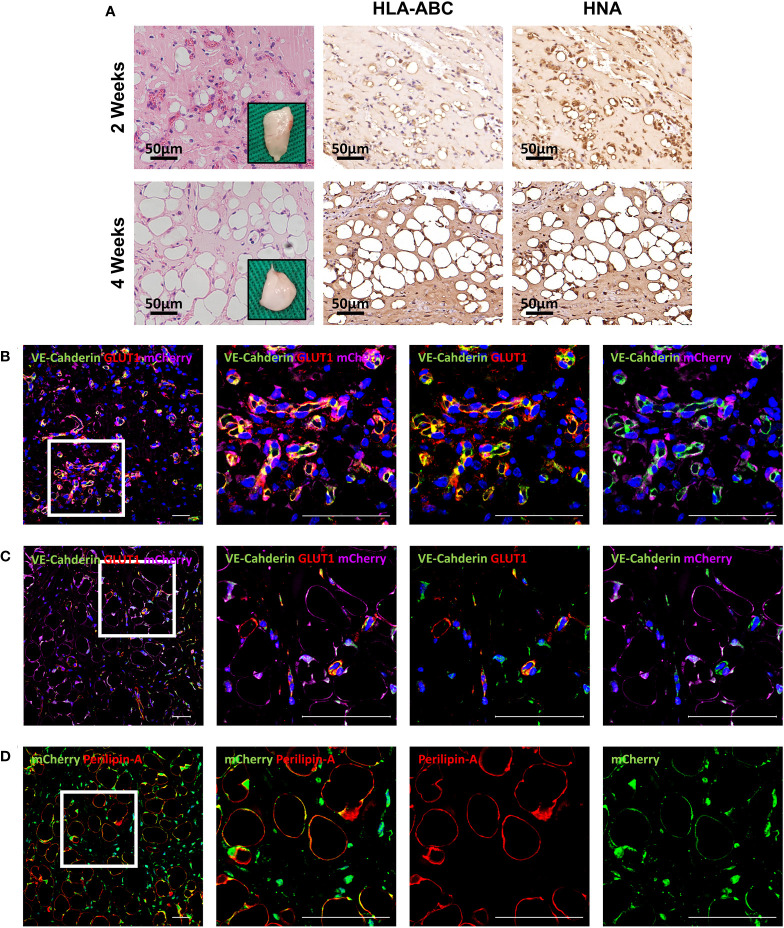
Combined injection of HemMCs and HUVECs leads to the formation of GLUT1^+^ vessels *in vivo*. **(A)** A mixture of HUVECs and HemMCs in Matrigel was injected into nude mice. Explants were harvested at 2 and 4 weeks and then subjected to H&E staining and IHC staining of HLA-ABC and HNA. Scale bars=50 μm. **(B-D)** A mixture of HUVECs and mCherry-labeled HemMCs in Matrigel was injected into nude mice. Short scale bars=50 μm; long scale bars=100 μm. **(B)** Explants were harvested at 2 weeks and then subjected to immunostaining for VE-cadherin (green), GLUT1 (red) and mCherry (magenta). **(C)** Explants were harvested at 4 weeks and then subjected to immunostaining for VE-cadherin (green), GLUT1 (red) and mCherry (magenta). **(D)** Explants were harvested at 4 weeks and then subjected to immunostaining for Perilipin-A (red) and mCherry (green). HemMCs, infantile hemangioma mural cells; HUVECs, human umbilical vein endothelial cells; GLUT1, glucose transporter 1; H&E, hematoxylin and eosin; IHC, immunohistochemistry; HLA-ABC, human leukocyte antigen-ABC. HNA, human nuclei antigen; VE-cadherin, vascular endothelial cadherin.

To assess the proangiogenic function of HemMCs *in vivo*, a mixture of Matrigel and two kinds of cells, i.e., HemMCs and HUVECs, were injected into immunodeficient nude mice. At 2 weeks, a considerable number of newly formed small vessels with blood cells were observed in the implants, and HLA-ABC and HNA immunostaining confirmed that these vessels were of human origin (**Figure 6A**). Immunofluorescence staining indicated that the vessels consisted of VE-Cahderin^+^GLUT1^+^ endothelial cells surrounded by mCherry-labeled HemMCs (**Figure 6B**). At 4 weeks, the number of vessels was significantly decreased (**Figure 6C**), while the number of mCherry^+^Perilipin-A^+^ adipocytes in the implants was increased (**Figure 6D**).

## 4 Discussion

In a previous study, it was found that pericytes and perivascular cells in IH lesions cannot be identified with a single pericyte marker; instead, extended characterization of 8 pericyte/smooth muscle cell markers and perivascular localization are needed to elucidate the specific phenotype of pericytes in IH lesions (10). However, hemangioma pericytes were previously isolated by a rough method, and proper markers for isolating perivascular cells have not been tested (5, 10); thus, other kinds of cells can also be present in adherent cultures. For example, fibroblasts share similar markers, including PDGFRβ, with pericytes, are also abundant in IH lesions and skin and easily adhere to uncoated culture dishes. In this study, we used anti-CD146 antibody-conjugated microbeads to isolate and concentrate CD31^-^CD146^+^ HemMCs from IH specimens for culture and further experiments. HemMCs exhibit phenotypic and functional characteristics of pericytes in IH tissues. Complementing the previous belief that endothelial cells derived from HemSCs are the main tumoral cells of hemangiomas (7), perivascular HemMCs also account for the majority of parenchymal cells in IH lesions. The number and proportion of HemMCs increase rapidly during the proliferation phase and gradually decrease in the involuting phase of IH.

Our study shows that HemMCs display mesenchymal stem cell-like properties, specifically multilineage differentiation potential, especially the ability to differentiate into adipocytes. Furthermore, we found that HemMCs spontaneously differentiated into adipocytes after subcutaneous implantation in combination with Matrigel into immunodeficient mice. This adipogenic feature of HemMCs is similar to that of HemSCs and GLUT1^+^ endothelial cells (20), relating to adipogenesis in the involuting process of IH. A previous study suggested that peroxisome proliferator activated receptor gamma (PPAR-γ), which is a key transcription factor in adipogenesis, is mainly expressed in perivascular cells and in a few endothelial cells in IH (22). These findings indicate that targeting the phenotype and functional transition of HemMCs probably plays an important role in accelerating involution in IH.

Rapid proliferation of IH is the result of dysregulation of both vasculogenesis and angiogenesis (1). HemSCs, which appear to be progenitor cells of IH, form functional blood vessels by differentiation into both ECs and pericytes, which is induced by angiogenic factors, such as vascular endothelial growth factor (VEGF) (23). This process is called vasculogenesis. The proliferation of endothelial cell and mural cells accounts for angiogenesis. Unlike retinal pericytes, Hem-pericytes can accelerate endothelial colony forming cell (ECFC) proliferation, migration, and VEGF-A secretion (10). We speculated that CD146-selective HemMCs play an important proangiogenic role in the growth phase of IH. Our study revealed that proangiogenic molecular pathways are enhanced in HemMCs and that HemMCs increase tube formation by HUVECs *in vitro*. Moreover, we found that HemMCs in combination with HUVECs can form VE-cadherin^+^ GLUT1^+^ microvessels, followed by Perilipin-A^+^ adipocytes *in vivo*, simulating the progression of IH. GLUT1 is expressed along the endothelium of hemangiomas in the proliferating and involuting phases. Immunostaining for GLUT1 can be used to distinguish IH lesions from other vascular tumors and vascular malformations (24, 25). In our murine xenograft model, the expression of GLUT1 in endothelial cells could be conditionally induced by HemMCs.

Implantation of HemSCs combined with Matrigel into immunodeficient mice is the most widely used method for studying HemSC-derived vasculogenesis in IH (7, 26, 27). VEGFR-1 mediates HemSC-to-EC differentiation *in vitro* and vessel formation in a HemSC/Matrigel xenograft model (23). However, Greenberger et al. proposed that this model is unstable and added endothelial cells such as HUVECs and cord blood endothelial progenitor cells (cbEPCs) (28–30). In addition, Boscolo et al. found that HemSCs can be induced to differentiate into pericytes by cbEPCs *via* JAGGED1 signaling regulation in this model (5).

Hem-pericytes cooperate with ECFCs to form CD31^+^ blood vessels when both are implanted into immunodeficient mice (10). Because GLUT1-positive HemECs are known to have the potential to differentiate into pericytes/smooth muscle cells (20), HUVECs were used in this study to identify the proangiogenic function of HemMCs. In this study, the CD146-selective HemMC/EC/Matrigel xenograft model represented pathological GLUT1^+^VE-cadherin^+^ microvessels surrounded by HemMCs, serving as a stable model for IH. In brief, the method used to establish this model is simple and standardized, as isolation of HemMCs with CD146-selective microbeads is efficient and repeatable. To reiterate, CD146-seletive HemMC should be added to IH models and used for research on the effectiveness of antiangiogenic or proangiogenic methods for the treatment of IH.

There are several differences between the HemMC/EC/Matrigel xenograft model and the HemSC/Matrigel xenograft model. First, the number of CD133-selective HemSCs in IH is very small, i.e., approximately 0.2% (7), and is hard to detect in stable IH, while the number of CD146-selective HemMCs is large. Second, HemMCs exhibit rapid proliferation and stronger proangiogenic ability than HemSCs; thus, the HemMC/EC/Matrigel model is more suitable for studying new therapies to treat involuting IH. Finally, HemMCs do not have endothelial differentiation potential; thus, the value of this model for studying vasculogenesis is limited.

In summary, HemMCs exhibit MSC-like features and proangiogenic ability *in vitro*, and a murine model established using HemMCs can simulate the pathological characteristics of IH. This study uses CD146-selective microbeads to isolate HemMCs, which surround the endothelium and proliferate quickly in IH. *In vitro*, HemMCs exhibit proangiogenic properties: they express markers of MSCs, exhibit strong angiogenesis-associated signaling, display multilineage differentiation potential, and enhance HUVEC tube formation. *In vivo*, CD146-selective HemMCs implanted with Matrigel spontaneously differentiate into adipocytes, while HemMCs implanted with HUVECs can form GLUT1-positive microvessels followed by Perilipin-A-positive adipose-like IH lesions. The proangiogenic function of HemMCs needs to be further studied to discover new drugs for the treatment of IH.

## Data availability statement

The data generated in this study has been deposited in NCBI’s Gene Expression Omnibus and is accessible through GEO Series accession number GSE216867 or at this link: https://www.ncbi.nlm.nih.gov/geo/query/acc.cgi?acc=GSE216867.

## Ethics statement

The studies involving human participants were reviewed and approved by the Ethics Committee of Shanghai Ninth People’s Hospital, Shanghai Jiao Tong University of Medicine. Written informed consent for participation was not required for this study in accordance with the national legislation and the institutional requirements. The animal study was reviewed and approved by the Ethics Committee of Shanghai Ninth People’s Hospital, Shanghai Jiao Tong University of Medicine.

## Author contributions

ZC and XL were the primary contributor to research design. JC, LC, YQ, YL, S-JC and QC were responsible for recruitment of patients, provision of human samples, research execution and were contributors to data acquisition. ZC and JC were the primary contributors to data analysis and interpretation. JC and ZC prepared the manuscript with revisions provided by ZC, XL, LC and YQ. ZC and XL were in charge of funding provision and study supervision. All authors contributed to the article and approved the submitted version.
