# Supplementary material for: CD146+ mural cells from infantile hemangioma display proangiogenic ability and adipogenesis potential in vitro and in xenograft models
Source: Front Oncol. 2023 Apr 27;13:1063673. doi: 10.3389/fonc.2023.1063673 (PMC10172585; doi:10.3389/fonc.2023.1063673)
Supplement: Supplementary file 1 [file DataSheet_1.docx]

Supplementary Material

# Supplementary Tables

## Infantile hemangioma specimens

| Number | Stage of IH | Location | Age | Sex |
| --- | --- | --- | --- | --- |
| 1 | Stable | Head | 8 months | Female |
| 2* | Proliferative | Head | 2 months | Female |
| 3* | Proliferative | Head | 4 months | Female |
| 4 | Stable | Forehead | 8 months | Female |
| 5* | Proliferative | Head | 4 months | Male |
| 6 | Involuting | Nose | 34 months | Female |
| 7 | Involuting | Shoulder | 28 months | Female |
| 8 | Involuting | Arm | 22 months | Male |
| 9 | Involuting | Nose | 4 years | Female |
| 10 | Proliferative | Lip | 4 months | Female |
| 11 | Involuting | Nose | 3 years | Female |
| 12 | Involuting | Lip | 3 years | Female |
| 13 | Proliferative | Arm | 4 months | Female |
| 14 | Proliferative | Head | 6 months | Female |
| 15 | Involuting | Abdomen | 26 months | Female |
| 16 | Involuting | Forehead | 24 months | Female |
| 17 | Involuting | Head | 15 months | Male |
| 18 | Proliferative | Abdomen | 5 months | Female |

**Supplementary Table 1.** **Information of infantile hemangioma specimens used in this study.** *Cells for culture were isolated from these specimens. Abbreviations: IH, infantile hemangioma.

## Antibodies used in this study

| Name of target | Company | Catalog Number | Applications |
| --- | --- | --- | --- |
| CD14 | BD Biosciences | 557923 | FCM |
| CD34 | BD Biosciences | 561440 | FCM |
| CD45 | BD Biosciences | 563792 | FCM |
| CD73 | BD Biosciences | 561260 | FCM |
| CD90 | BD Biosciences | 563070 | FCM |
| CD105 | BD Biosciences | 563920 | FCM |
| CD140b | BD Biosciences | 558821 | FCM |
| CD146 | BD Biosciences | 563619 | FCM |
| CD146 | Abcam | ab75769 | IF |
| CD31 | Abcam | ab275989 | IF |
| PDGFRβ | Cell Signaling Technology | 3169 | IF |
| GLUT1 | Abcam | ab115730 | IF |
| VE-Cadherin | R&D Systems | AF938 | IF |
| GFAP | Millipore | MAB360 | IF |
| β-tubulin III | Abcam | ab78078 | IF |
| mCherry | Abcam | ab213511 | IF |
| perilipin-A | Cell Signaling Technology | 9349 | IF |
| HLA-ABC | Santa Cruz Biotechnology | sc-271388 | IHC |
| HNA | Abcam | ab190710 | IHC |
| HRP-Polymer anti-Mouse IgG | MaxVision | KIT-5001 | IHC |
| Alexa Fluor^TM^ PIus 488 Donkey anti-Mouse IgG | Thermofisher | A32766 | IF |
| Alexa Fluor^TM^ PIus 488 Donkey anti-Rabbit IgG | Thermofisher | A32790 | IF |
| Alexa Fluor^TM^ PIus 555 Donkey anti-Mouse IgG | Thermofisher | A32773 | IF |
| Alexa Fluor^TM^ PIus 555 Donkey anti-Rabbit IgG | Thermofisher | A32794 | IF |
| Alexa Fluor™ 647 Donkey anti-Goat IgG | Thermofisher | A21447 | IF |

## Supplementary Table 2. Information of antibodies used in this study. Abbreviations: FCM, flow cytometry; IF, immunofluorescence; PDGFRβ, platelet-derived growth factor receptor beta; GLUT1, glucose transporter 1; VE-cadherin, vascular endothelial cadherin; GFAP, glial fibrillar acidic protein; HLA-ABC, human leukocyte antigen-ABC. HNA, human nuclei antigen; IHC, immunohistochemistry.

# Supplementary Figures

## Supplementary Figure 1


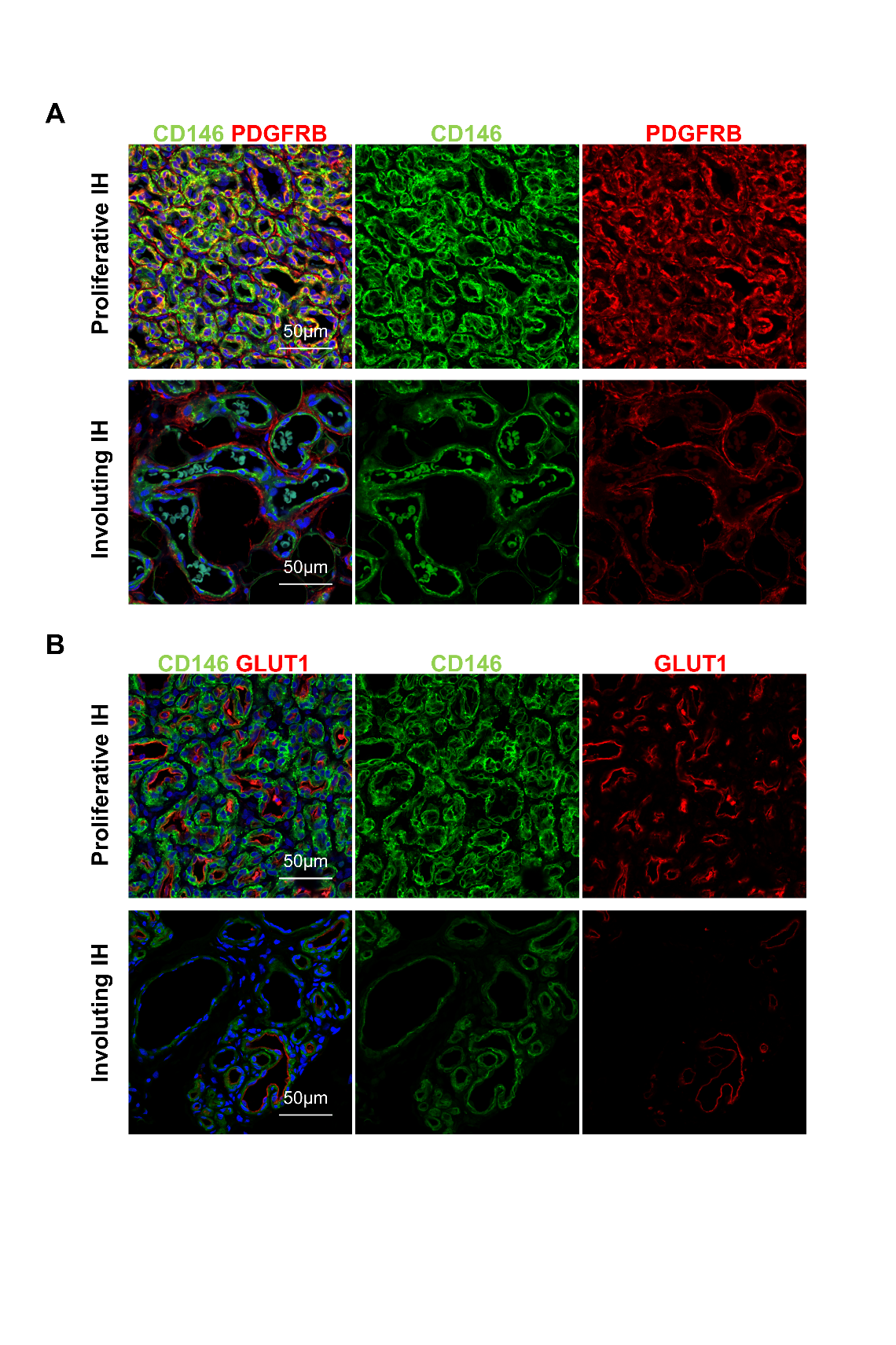


**Supplementary Figure 1.** **CD146 expression in proliferative and involuting IH tissue.** A representative proliferative IH specimen was collected from a 2-month-old patient with rapid growth hemangioma. A representative involuting IH specimen was collected from a 3-year-old patient with involuted hemangioma. (A) Representative images of IH specimens stained for CD146 (green) and PDGFRβ (red). (B) Representative images of IH specimens stained for CD146 (green) and GLUT1 (red). Scale bars=50 μm. All nuclei were counterstained blue (DAPI). Abbreviations: IH, infantile hemangioma;PDGFRβ, platelet-derived growth factor receptor beta; GLUT1, glucose transporter 1.

## Supplementary Figure 2


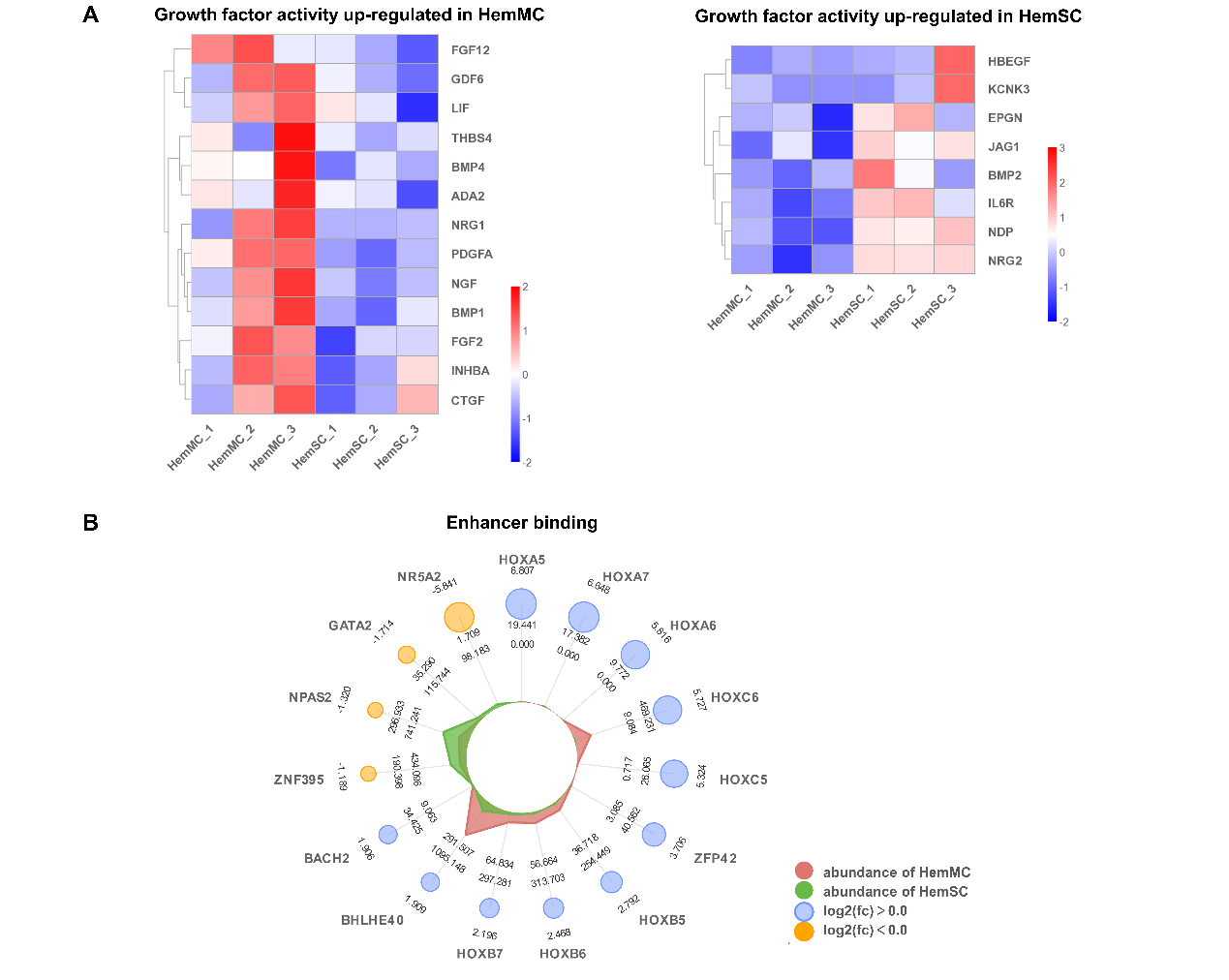


**Supplemental Figure 2. GO terms of growth factor activity and enhancer binding.** (A) Heatmap showing the differential expression of HemMCs and HemSCs enriched in growth factor activity (GO: 0008083). (B) Radar plot showing the differential expression of HemMCs and HemSCs enriched in enhancer binding (GO: 0035326). P-value <0.05. Abbreviations: HemMCs, infantile hemangioma mural cells; HemSCs, infantile hemangioma stem cells; GO, gene ontology.
